# Pharmacological Management of Anxiety in End‐of‐Life Care: A Systematic Review of Benzodiazepines, Opioids, and Psilocybin

**DOI:** 10.1002/hup.70032

**Published:** 2026-01-07

**Authors:** Brunno Freitas da Costa, Paula Hartmann, Daniel Pagnin

**Affiliations:** ^1^ Antônio Pedro University Hospital Fluminense Federal University Niterói Brazil; ^2^ Institute of Biomedical Sciences Rio de Janeiro Federal University Rio de Janeiro Brazil; ^3^ Department of Psychiatry and Mental Health Faculty of Medicine Fluminense Federal University Niterói Brazil

**Keywords:** anxiety, benzodiazepines, end‐of‐life care, opioids, psilocybin

## Abstract

**Objective:**

Anxiety is common in patients receiving end‐of‐life care and significantly impacts their quality of life. However, pharmacological management remains challenging due to complex clinical presentations and potential side effects, emphasizing the need for systematically reviewing existing treatments. Here we aim to systematically evaluate the efficacy and safety of pharmacological treatments for anxiety in end‐of‐life care.

**Design:**

Systematic review following PRISMA guidelines, prospectively registered in PROSPERO (CRD42024556913). Comprehensive searches were performed in PubMed, Embase, Cochrane Library, and ClinicalTrials.gov. Eligible studies included adults receiving end‐of‐life care and evaluated pharmacological interventions targeting anxiety.

**Results:**

Five studies met inclusion criteria: two assessing benzodiazepines combined with opioids and three evaluating psilocybin. Both benzodiazepine‐opioid combinations and psilocybin reduced anxiety symptoms. Psilocybin studies reported rapid and sustained anxiety relief, with approximately 60%–80% of participants experiencing clinically significant improvements. Both treatment categories showed good tolerability without serious adverse events. However, the evidence base was limited by small sample sizes and narrow study contexts.

**Conclusions:**

Benzodiazepine‐opioid combinations and psilocybin show promise for anxiety relief in end‐of‐life patients. Nevertheless, limited high‐quality evidence highlights an important research gap. Further robust clinical trials are needed to confirm these findings and guide clinical practice in palliative care.

## 1 Introduction

The ‘end‐of‐life’ period is characterized by a progressive life‐limiting disease with a prognosis of months or less (Hui et al. 2014). This phase is marked by unique challenges, with patients often experiencing psychological distress, including death anxiety and existential suffering, which can significantly impact their quality of life (Hong et al. 2022).

Anxiety is a common condition among patients receiving end‐of‐life care, often exacerbated by the prognosis and the experience of dealing with a terminal illness. Studies indicate that up to 48% of patients in palliative care report significant anxiety symptoms (Stark et al. 2002), which can negatively impact quality of life and complicate the management of physical symptoms (Mystakidou et al. 2005; Stark et al. 2002). Effective recognition and treatment of anxiety are thus crucial for providing adequate and patient‐centered care (Bookbinder and McHugh 2010).

Pharmacological treatment of anxiety in end‐of‐life care presents distinctive challenges due to the complexity of the patient's conditions and the need to minimize unwanted side effects. Commonly used medications include benzodiazepines, antidepressants, also with reports of use of antipsychotics, opioids and stimulant drugs, yet the selection of specific agents must be carefully tailored to the individual patient's clinical condition and preferences (Rayner et al. 2010). Furthermore, emerging evidence suggests that psilocybin may offer a promising alternative to traditional anxiolytics, with some studies demonstrating rapid and sustained reductions in anxiety and depression, as well as a favorable safety and tolerability profile (Haikazian et al. 2023). This body of established and preliminary findings has contributed to its growing role in the management of anxiety in end‐of‐life care. Despite its promising efficacy and tolerability, the administration and access to psilocybin remain constrained by regulatory restrictions and logistical challenges (Behera et al. 2024). The efficacy and safety of these treatments are areas of ongoing interest, as side effects can exacerbate other symptoms or reduce the patient's functional status (Bookbinder and McHugh 2010).

In addition to clinical challenges, there are significant barriers to the identification and treatment of anxiety in patients at the end of life. Factors such as underreporting of symptoms due to stigma, healthcare professional's difficulty in recognizing signs of anxiety, and the often‐prioritized management of physical pain can lead to the undertreatment of this condition (Borges et al. 2017). It is essential that palliative care integrates a multidisciplinary approach that addresses both the physical and psychological aspects of patient suffering (Zimmermann et al. 2014). Zimmermann et al. (2014) found that early palliative care delivered by a coordinated multidisciplinary team significantly improved both physical symptom management and psychological well‐being in patients with advanced cancer.

Given these challenges in recognizing and treating anxiety at the end of life, there is a clear need for a comprehensive synthesis of available pharmacological interventions. Although previous meta‐analyses (Yu et al. 2021) have examined emerging treatments like psilocybin, our review extends this work by also evaluating conventional approaches—including benzodiazepines, opioids, and other agents—within a single framework. This integrative perspective provides a more complete overview of the therapeutic landscape for managing anxiety in terminally ill patients and highlights critical gaps in the current evidence base that call for further research.

In this study we aim to conduct a systematic review to analyze the current pharmacological treatment strategies for anxiety in adult patients receiving end‐of‐life care. We will examine the effectiveness of interventions, explore potential barriers to the recognition and treatment of anxiety, and identify areas for further research and improvement in the management of anxiety in this population.

## 2 Methods

This systematic review was registered in PROSPERO (CRD42024556913) and aims to investigate the current pharmacological treatment strategies for anxiety in adult patients receiving end‐of‐life care.

### 2.1 Scope of the Study

The inclusion criteria were studies involving adult patients (18 years or older) receiving end‐of‐life care due to terminal conditions, including cancer, heart failure, advanced neurological diseases or other terminal conditions. Eligible studies evaluated the efficacy and/or safety of any pharmacological intervention for managing anxiety in these patients, included a comparator, such as placebo, another anxiolytic medication, or usual care, and reported at least one outcome related to the efficacy or safety of the intervention. Suitable study designs included randomized controlled trials (RCTs), non‐randomized controlled studies, cohort studies, and case series providing comparative information.

The exclusion criteria for this review were studies that focused on pediatric patients or those that did not clearly differentiate between general palliative care and end‐of‐life care. Studies exclusively centered on populations with primary mental health conditions, non‐pharmacological interventions, or those that solely addressed other symptoms like pain or depression without focusing on anxiety were also excluded. Specifically, we defined ‘primary mental health conditions' as those in which the main focus of treatment was chronic psychiatric disorders—such as recurring depression, severe anorexia leading to organ failure, or other conditions where the mental illness was the principal driver of the terminal status—rather than anxiety secondary to a terminal physical illness. Consequently, studies in which patients were treated primarily for long‐standing mental health issues, even if they also had a terminal illness, were not considered eligible. Additionally, studies lacking a comparator, single‐case studies, narrative reviews, editorials, and letters to the editor were excluded from the review.

### 2.2 Search Process

To identify relevant studies, comprehensive searches were conducted in electronic databases including PubMed (at 12/06/2024), EMBASE (at 12/06/2024), Cochrane Library (at 16/06/2024) and ClínicalTrials.gov (at 24/06/2024) using the following search terms.

- “End of life care” OR “Terminal care” OR “Hospice care” OR “Terminally ill” OR “End‐stage disease” OR “Advanced disease”
- “Anxiety” OR “anxiousness” OR “nervousness” OR “panic” OR “fear” OR “worry”
  - AND (“Pharmacological treatment” OR “Drug therapy” OR “Medication” OR “Pharmacotherapy” OR “Benzodiazepines” OR “Antidepressants” OR “SSRIs” OR “SNRIs” OR “MAOIs” OR “Tricyclic antidepressants” OR “Antipsychotics” OR “Anxiolytics” OR “Sedatives” OR “Tranquilizers”)
- “Placebo” OR “Usual care” OR “standard care” OR “Control group” OR “Comparative”
- “Efficacy” OR “Effectiveness” OR “Outcomes” OR “Symptom relief” OR “Symptom reduction” OR “Quality of life” OR “QOL” OR “Side effects” OR “Adverse effects” OR “Safety”

Specific approaches were used for each search tool (Supporting Information S1: Appendix).

We also hand searched the reference lists of relevant studies, particularly those from previously published systematic reviews in related fields. This strategy aimed to identify additional articles that might have been missed in our initial database searches.

The literature search was limited to published original articles written in English language. No date limits were used.

### 2.3 Selection, Data Extraction and Bias Assessment

The selection process for our systematic review began with one researcher extracting reports from MEDLINE via PubMed, EMBASE, Cochrane Library and ClinicalTrials. No automation tools were used beside Zotero's duplicate identifier. Titles and abstracts were screened manually to exclude irrelevant subjects.

Next, two independent researchers assessed the remaining reports for eligibility. Discrepancies were resolved through discussion, and if necessary, a third researcher was consulted to reach a consensus. This thorough process ensured the inclusion of relevant studies.

Data from the selected articles were collected by one researcher, and the results of this collection were reviewed and supervised by two additional researchers. The interventions were divided into two groups due to their similarities.

Bias assessment was conducted using RoB‐2 (Sterne et al. 2019) for randomized trials, RoB‐2 adapted for crossover trials and ROBINS‐I (Sterne et al. 2016) for non‐randomized studies in order to ensure the inclusion of relevant and high‐quality studies.

## 3 Results

### 3.1 Study Selection

**FIGURE 1:**
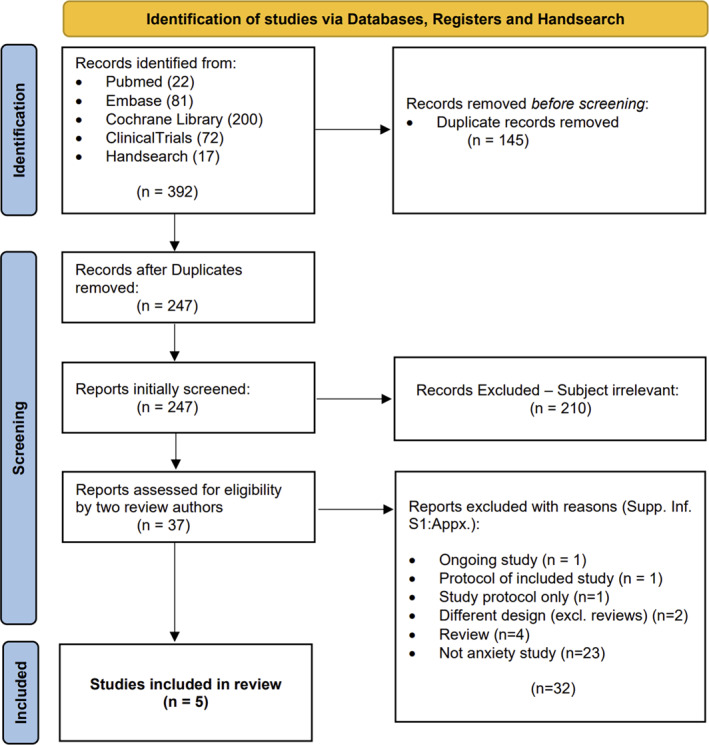
Study selection diagram. PRISMA Flow Diagram (Page et al. 2021) explaining the research and selection process.

A total of 5 studies were included in this review (Figure 1). The systematic database search resulted in 392 records identified with 145 being removed before screening for being duplicates.

In the initial screening, 247 studies were analyzed for its title and abstract. After removing 210 studies with irrelevant subjects, 37 reports were assessed for eligibility by two independent review authors. Following the exclusion of 31 studies that did not fulfill our eligibility criteria (reasons of exclusion explained in Supporting Information S1: Appendix), 6 studies remained for inclusion. One of them was identified to be an ongoing study after contacting the authors, with the recruitment already completed but no data available (Atkin et al. 2023).

### 3.2 Study Characteristics

#### 3.2.1 Methods

Among the included studies, one had three randomized arms, each receiving different interventions. Another study used a design that compared the effect of the intervention to the same group at baseline. One study was conducted as a double‐blind, randomized clinical trial. Additionally, two studies employed a crossover design.

#### 3.2.2 Participants

All the studies explicitly stated that the participants had terminal, life‐threatening illnesses, with some participants passing away during the course of the studies. There was no measure of prognosis or life expectancy.

#### 3.2.3 Intervention

Two of the studies used a combination of benzodiazepines and opioids as the intervention. In Navigante et al. (2006), groups received fixed Morphine (2.5 mg, 4‐4h) with Midazolam rescues (5 mg), fixed Midazolam (5 mg, 4‐4h) with Morphine rescues (2.5 mg) or fixed Morphine (2.5 mg, 4‐4h) plus Midazolam (5 mg, 4‐4h). Whereas Clemens and Klaschik (2011) used Morphine/Hydromorphone plus Lorazepam (every 4h, with opioid dose chosen depending on dyspnea intensity and performance status).

The other three studies involved the use of psilocybin as an intervention for the potential treatment of anxiety. The dosage in each study was calculated based on the patient's weight (Table 1).

**TABLE 1:** Selected studies.

	Study design	Sample size	Participants	Intervention	Comparator	Co‐intervention	Primary outcome	Secondary outcome	Follow‐up
Navigante et al. (2006)	Randomized, controlled study	101	Documented diagnosis of terminal advanced cancer, life expectancy less than a week	Fixed morphine (2.5 mg 4/4h) with midazolam rescues (5 mg)	Fixed midazolam (5 mg 4/4h) with morphine rescues (2.5 mg); Fixed morphine (2.5 mg 4/4h) plus midazolam (5 mg 4‐4h)	—	Modified BPotentiorg scale (intensity of dyspnea)	The national cancer institute common toxicity criteria (CTC)	—
Clemens and Klaschik (2011)	Prospective non‐randomized study	26	Terminal stage of a cancer or other incurable diseases	Morphine/Hydromorphone + lorazepam	Baseline X 30, 60, 90 and 120 min after the first opioid and anxiolytics application	Nonpharmacological support (breathing therapy, relaxation exercise)	Dyspnea NRS 0–10, paCO2, SaO2	Pulse frequency, respiratory frequency	—
Grob et al. (2011)	Double‐blind, placebo‐controlled study	12	Advanced‐stage cancer, two subjects died of their cancer during the follow‐up period	Psilocybin (0.2 mg/kg) (crossover)	Niacin (250 mg) (crossover)	—	BDI, POMS, STAI	5D‐ASC, BPRS	Monthly for 6 months
Ross et al. (2016)	Randomized, blinded, placebo‐controlled, crossover study	29	Nearly two‐thirds of participants (62%) had advanced cancers (stages III or IV)	Psilocybin (0.3 mg/kg) (crossover)	Niacin (250 mg) (crossover)	Psychotherapy	HADS, BDI, STAI	HAI, DAS, DTS, WHO‐bref, FACIT‐SWB	2, 6, 7, 26 weeks
Griffiths et al. (2016)	Randomized, blinded, placebo‐controlled, crossover study	51	Potentially life‐threatening cancer diagnosis, with 65% having recurrent or metastatic disease.	High psilocybin dose (22 or 30 mg/70 kg) (crossover)	Low psilocybin dose (1 or 3 mg/70 kg) administered in identically appearing capsules (crossover)	—	Depression: GRID‐HAM‐D‐17 and anxiety: HAM‐A/SIGH‐A	Fifteen secondary measures focused on psychiatric symptoms, moods, and attitudes	5 weeks and 6 months

*Note:* Table with the selected studies characteristics.

Abbreviations: 5D‐ASC = Five Dimensional Altered States of Consciousness; BPRS = Brief Psychiatry Rating Scale; BDI = Beck's Depression Inventory; DAS = Death anxiety scale; DTS = Distress Tolerance Scale; FACIT = Functional Assessment of Chronic Illness Therapy; HADS = Hospital Anxiety and Depression Scale; HAM‐D = Hamilton Depression Rating Scale; HAM‐A = Hamilton Anxiety Rating Scale; NRS = Numerical rating scale; POMS = The Profile of Mood States; STAI = Stait‐Trait Anxiety Scale; SWB = Subjective Wellbeing Scale.

#### 3.2.4 Outcomes

There was heterogeneity among the outcome measures used in the selected studies. Two of the studies focused on the assessment of dyspnea, using the Modified Borg scale and the Dyspnea NRS scale, with one of them explicitly detailing the correlation between dyspnea and anxiety. In the other studies, more classical scales for assessing anxiety, such as the STAI and HAM‐A, were used.

### 3.3 Risk of Bias in Studies

To assess the quality and risk of bias of the selected studies, tools appropriate to the design of each study were used to evaluate their specific characteristics as accurately as possible.

**FIGURE 2:**
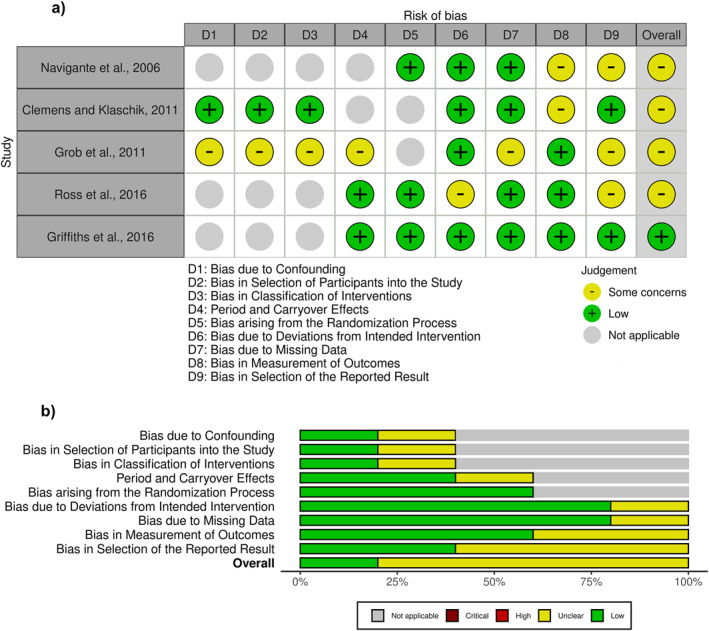
Risk of bias in selected studies. Risk of bias analysis using RoB‐2, Robins‐i and RoB‐2 adapted for crossover trials and Robvis (McGuinness and Higgins 2021); (a) Traffic light plot; (b) Summary plot; Not applicable ‐ dimension not assessed due to study design.

The study by Navigante et al. (2006) despite having randomized comparison groups and representing the largest sample size among the selected studies, raised some concerns, especially because it is a single‐blinded study where both the intervention administrator and the outcome assessors were aware of the interventions performed, which could introduce some risk of bias into the results. Additionally, there was no information in the paper or its registration about the planned statistical analyses (Figure 2).

In the study by Clemens and Klaschik (2011), in addition to the absence of randomization and lack of group division for better comparison, the fact that the outcome assessors were aware of the intervention also raises concerns, introducing concerns about a risk of bias to the study (Figure 2).

In the pilot study published by Grob et al. (2011) the small sample size is noteworthy. Participants acted as their own control, meaning that it lacks a control group and randomization. It was not clarified how much time elapsed between the first and second intervention sessions, raising the possibility of a carry‐over effect. The results were not reported numerically, only graphically. Additionally, measures of dispersion were not provided. We contacted the authors but were unable to obtain additional data. These factors raise concerns about the risk of bias in the selection of the presented results. Furthermore, there was also no information about prior plans for statistical analysis (Figure 2).

Ross et al. (2016) was a double‐blind study. However, the information contained in the supplementary material suggests that the psychoactive effects characteristic of the intervention may have compromised ideal blinding in the study, leading to some concern about bias in the results (Figure 2).

In the study by Griffiths et al. (2016), a control group that also received the psychoactive substance, but at a lower dose, was used. This approach appears to have ensured greater reliability in the results (Figure 2).

### 3.4 Results of Individual Studies

#### 3.4.1 Opioids and Benzodiazepines Studies

Two studies analyzed the use of opioids in combination with benzodiazepines for managing dyspnea associated with anxiety in end‐of‐life patients. Both studies demonstrated positive results in terms of efficacy for anxiety control and safety of these medications.

In the study by Clemens and Klaschik (2011), 26 patients with moderate to severe dyspnea associated with anxiety were treated with opioids (morphine or hydromorphone) in combination with lorazepam. The results showed a significant reduction in dyspnea intensity both at rest and during exertion after 120 min of treatment (Table 2). Importantly, the treatment did not lead to any signs of respiratory depression, even in hypoxemic patients, as oxygen saturation (from 95.0 ± 4.6 to 95.2 ± 3.5 after 120 min) and carbon dioxide levels (from 38.1 ± 6.0 to 37.7 ± 5.5 after 120 min) generally remained stable.

**TABLE 2:** Outcomes of included studies.

Study	Anxiety measure	Results								
**Opioids and benzodiazepines studies**										
Navigante et al. (2006)	Modified borg scale	Baseline			24h			48 h		
		Mo	Mi	MM	Mo	Mi	MM	Mo	Mi	MM
		7.1 ± 0.8*	6.9 ± 1.0*	6.8 ± 0.8*	3 (2–5.5)**	4 (2–6.2)**	3 (2–5)**	2 (0–4.7)**	2 (0–7)**	2 (1–5)**
Clemens and klaschik (2011)	Dyspnea NRS		Baseline	30 min	60 min	90 min	120 min			
		At rest Exertion	6.2 ± 2.2* 7.4 ± 2.3*	4.1 ± 2.0* 5.5 ± 3.0*	2.4 ± 1.5* 3.4 ± 1.7*	1.4 ± 1.0* 2.7 ± 1.3*	1.2 ± 0.8* 2.5 ± 1.2*			
**Psilocybin studies**										
Grob et al. (2011)	STAI	Not clear								
Ross et al. (2016)	STAI		1 day	2 weeks	6 weeks	7 weeks				
		State	*d* = 1.20	*d* = 1.45	*d* = 1.27	*d* = 1.18				
		Trait	*d* = 0.95	*d* = 1.49	*d* = 1.31	*d* = 1.29				
Griffiths et al. (2016)	HAM‐A			Baseline		Post‐session 1		Post‐session 2	6 months	
		Low dose 1st (High dose 2nd)		25.68 (0.89)***		16.64 (1.53)***		8.92 (1.14)***	7.95 (1.19)***	
		High dose 1st (low dose 2nd)		25.73 (1.11)***		8.48 (1.16)***		7.52 (1.27)***	7.04 (1.17)***	

Abbreviations: *d* = Cohens'd; Mi = Midazolam with Morphine rescues, MM = Morphine plus Midazolam; Mo = Morphine with Midazolam rescues; * = Mean ± Standard deviation; ** = Median (interquartile range); *** = Mean (standard error mean).

Similarly, in the study by Navigante et al. (2006), 101 patients with advanced cancer and severe dyspnea were divided into three groups. The group receiving the combined therapy of fixed Morphine plus Midazolam showed the best outcomes for dyspnea relief, with 92% of patients reporting improvement after 24 h, compared to 69% in the Morphine alone with Midazolam rescues group and 46% in the Midazolam alone with Morphine rescues group. Additionally, only 4% of patients in the combination group had unresolved dyspnea after 48 h and there were fewer breakthrough dyspnea episodes in this group. In terms of side effects, clinically relevant adverse events were minimal, and the combination therapy caused less sedation than morphine alone (9% × 17%). There were no cases of severe respiratory depression.

Both studies highlight the effectiveness of combining opioids with benzodiazepines for the management of anxiety and dyspnea in end‐of‐life patients. The findings suggest that this classical approach is safe, with few serious side effects reported, and remain a valuable therapeutic option for symptom management in end‐of‐life care.

#### 3.4.2 Psilocybin Studies

The other three studies examined the use of the psychedelic compound psilocybin in the treatment of anxiety and depression in terminally ill patients.

In Grob et al. (2011), 12 participants were enrolled. The study found a sustained and statistically significant reduction in trait anxiety, as measured by the STAI scale, at the 1‐month and 3‐month follow‐up assessments after the intervention. There were no serious adverse events during or after the psilocybin sessions. The transient physiological effects, such as mild increases in blood pressure and heart rate, were short‐lived and manageable, with no long‐term complications observed.

The study by Ross et al. (2016), which included 29 participants, further supported those findings. It showed that patients experienced quick and long‐lasting decreases in anxiety after a single dose of psilocybin (Table 2). At the 6.5‐month follow‐up, 60%–80% of participants continued to have clinically meaningful reductions in anxiety. The study also found significant improvements in existential distress, spiritual well‐being, and quality of life. Participants reported reduced feelings of cancer‐related demoralization and hopelessness and exhibited significantly improved attitudes and adaptations toward death at the 6.5‐month follow‐up. As in the Grob et al. (2011) study, no severe adverse events were reported. The physiological effects, such as transient increases in systolic blood pressure and heart rate were mild and did not pose any lasting health risks, with peak mean systolic and diastolic blood pressure of 142/83mmHg recorded at 180 min post‐dosing.

Similarly, the study by Griffiths et al. (2016) found that psilocybin led to significant reductions in anxiety. After the first dose, 76% of participants in the High‐dose group had Clinical response versus 24% in Low‐dose Group (*p* < 0.001). Approximately 80% of participants had clinical response at the 6‐month follow‐up with reductions of more than 50% in symptom severity. Furthermore, the study found large significant changes in a therapeutically relevant direction (decreases in negative affect and increases in positive attitudes about death and life meaning and coherence) from Baseline to 6‐Month Follow‐up with mean effect size of 1.35. The intervention was well‐tolerated, with no serious adverse events and only slight and transient increases in blood pressure and heart rate during the psilocybin sessions.

These studies indicate that psilocybin may effectively treat anxiety in patients with life‐threatening illnesses. The consistent outcomes across studies highlight psilocybin's therapeutic potential, with significant reductions in anxiety lasting for months. Additionally, psilocybin has a favorable safety profile, with no severe adverse events and only mild, temporary physiological effects during administration (Griffiths et al. 2016; Ross et al. 2016). These findings suggest that psilocybin, when provided in a controlled and supportive environment, can offer rapid and lasting relief for patients experiencing existential distress at the end of life.

Regarding side effects that usually may raise concern when talking about psychedelic substances, the studies reported that psilocybin was generally well‐tolerated. Mild nausea was observed in 14% of individuals, and a smaller proportion (7%) experienced brief and transient psychosis‐like symptoms during the sessions (Ross et al. 2016). Importantly, no long‐term cardiovascular or psychiatric problems were noted, suggesting that psilocybin can be safely administered in a controlled, supportive setting.

## 4 Discussion

The concept of “end of life” is inconsistently defined across the literature (Hui et al. 2014), which complicates the discussion of treatment strategies within this context. Beyond the variability in terminology, there is a persistent challenge in accurately determining a patient's prognosis and life expectancy. This uncertainty can profoundly affect decision‐making and the management of care, often leading to divergent approaches in treatment (Martin and Widera 2020).

An additional complexity arises in the accurate identification of anxiety symptoms in patients approaching the end‐of‐life stage. These symptoms frequently overlap with physical manifestations, such as dyspnea, and are often misattributed solely to organic causes (Roth and Massie 2007). The tendency to neglect the psychological aspects of symptoms may worsen both dyspnea and pain, emphasizing the importance of a comprehensive evaluation (Kemp 1997).

In this review, two studies specifically used dyspnea as a primary outcome, with one of them highlighting a direct correlation between anxiety and the severity of dyspnea. The data suggest that anxiety may be a response to an initial experience of breathlessness, and that anxiety levels were significantly associated with the severity of dyspnea (Clemens and Klaschik 2011). This demonstrates the intricate relationship between mental and physical health in this population.

When considering the pharmacological treatment of anxiety, antidepressants are generally the first‐line therapy for the broader population (Katzman et al. 2014). However, their delayed onset of action significantly limits their utility in end‐of‐life patients, who require more immediate symptom relief. This creates a unique challenge in this clinical setting, where time is often a critical factor in the choice of therapy.

### 4.1 Main Findings/Results of the Study

Traditionally, benzodiazepines and opioids have been employed to manage anxiety in end‐of‐life care, especially within the context of palliative sedation (Slawnych 2020). Despite their common usage, only two studies met the inclusion criteria for this review, highlighting the scarcity of research focused specifically on anxiety management in this setting with these agents. This gap points to a pressing need for more targeted investigations into their efficacy and safety, particularly for the management of anxiety symptoms. Both studies demonstrated consistent symptom relief with minimal adverse effects, suggesting these medications remain valuable therapeutic options.

In contrast to these conventional treatments, psychedelic substances, particularly psilocybin, have emerged in the last decade as promising alternatives in various psychiatric fields, including the treatment of depression and post‐traumatic stress disorder (Nutt 2019). In this review, psilocybin surfaced as a potential treatment option for anxiety in end‐of‐life care, offering a novel and potentially transformative approach to therapy in this context.

Psilocybin's appeal is further enhanced by its rapid onset of action and its apparently favorable safety profile (Whinkin et al. 2023), making it especially suitable for patients in end‐of‐life care who require fast and effective relief from anxiety. These characteristics differentiate it from traditional medications like antidepressants, which often take weeks to demonstrate efficacy.

Three of the studies selected in this systematic review reveal a convergence of data supporting the use of psilocybin in reducing anxiety in end‐of‐life care. These findings suggest a promising and consistent therapeutic potential for psilocybin, even with a small number of studies available, and highlight the need for further investigation in this area.

However, despite the encouraging results surrounding psilocybin, further robust clinical trials are essential to firmly establish its efficacy and safety in end‐of‐life anxiety treatment. The evidence remains preliminary and larger, well‐designed studies are needed to validate its therapeutic potential and guide clinical practice.

One notable challenge in psychedelic research, including psilocybin trials, is the difficulty in achieving effective blinding, which raises concerns about potential bias in the reported outcomes (Van Elk and Fried 2023). This methodological issue has been a point of concern in psychedelic research more broadly, and it underscores the need for innovative trial designs to ensure rigorous evaluation of these treatments.

The risk‐of‐bias assessments across the included studies showed significant methodological limitations, such as insufficient blinding, small sample sizes, and inconsistent reporting practices. These factors may lead to an overestimation of treatment effects, thus necessitating a cautious interpretation of the findings. Furthermore, the study by Grob et al. (2011) did not provide clear numerical data for its anxiety outcomes, relying instead on graphical representations, which adds another layer of uncertainty to our synthesis. Overall, while the convergence of results is encouraging, these issues highlight the need for more robust, well‐designed trials to firmly establish the efficacy and safety of these interventions.

### 4.2 Potential Clinical Implications Based on Current Evidence

Although the current evidence base is limited, the findings from this review offer some guidance for clinical decision‐making in end‐of‐life care, suggesting a potential benefit in stratifying treatment according to the predominant clinical presentation.

For anxiety that is closely intertwined with physical symptoms—particularly dyspnea—the studies by Navigante et al. (2006) and Clemens & Klaschik (2011) suggest that the concurrent administration of opioids (such as morphine) and benzodiazepines (e.g., midazolam or lorazepam) appears to be a viable and generally well‐tolerated strategy. Despite common concerns regarding respiratory depression, the reviewed data indicate that, when carefully titrated and monitored, this combination may alleviate dyspnea‐associated anxiety without significant compromise to oxygen saturation. Clinicians might consider this approach particularly for patients where ‘air hunger' exacerbates anxious distress.

Conversely, for anxiety characterized primarily by existential distress, demoralization, and fear of death—symptoms often less responsive to traditional anxiolytics (Niles et al. 2021)—preliminary evidence suggests psilocybin could be a promising alternative. The studies reviewed indicate that psilocybin may facilitate rapid and sustained symptomatic relief. While regulatory frameworks currently limit its routine use, these findings support the consideration of psilocybin within the context of clinical trials or, where legal structures permit, compassionate use programs for patients with refractory existential suffering.

### 4.3 Strengths and Weaknesses/Limitations of the Study

Our review presents important strengths and limitations that must be considered. A notable strength is the rigorous methodology, including adherence to PRISMA guidelines and prospective registration on PROSPERO, ensuring transparency and reproducibility. However, several limitations are also evident. The limited number of included studies, with relatively small sample sizes, may reduce the robustness and generalizability of the conclusions. Additionally, restricting the search to English‐language publications might have led to exclusion of relevant studies published in other languages. Furthermore, the psilocybin studies identified originated exclusively from the United States (Table 3) and predominantly included cancer patients, which may limit generalizability to other terminal conditions, such as advanced heart failure or neurological diseases, and to diverse cultural contexts. Lastly, regulatory and legal restrictions surrounding psilocybin pose significant challenges for the broader implementation of this treatment in clinical practice, highlighting the need for further context‐sensitive studies.

**TABLE 3:** Country of origin, participant demographics, and funding source.

Study	Country	Age	Female	Education	Funding source
Navigante et al. (2006)	Argentina	57.3 (Mean)	53.4%	Not reported	Angel H. Roffo cancer institute
Clemens and klaschik (2011)	Germany	66 (Mean)	46.2%	Not reported	Deutschen krebshilfe
Grob et al. (2011)	USA	36–58 (Range)	91.6%	Not reported	NIH grant, heffter research institute; the betsy gordon foundation; the nathan Cummings foundation
Ross et al. (2016)	USA	56.3 (Mean)	62%	3% grade 7–12 3% high school 14% part‐college 79% college	Heffter research institute, the RiverStyx foundation, the New York University‐Health and Hospitals Corporation (NYU‐HHC) clinical and translational science institute (CTSI)
Griffiths et al. (2016)	USA	56.3 (Mean)	49%	2% high school 45% college 53% post‐graduate	NIH grant, heffter research institute, the riverstyx foundation, william linton, the betsy gordon foundation, the McCormick family, the fetzer institute, george goldsmith and ekaterina malievskaia

*Note:* “Not reported” indicates that the study did not provide sufficient information in the publication to determine this characteristic.

Abbreviation: NIH – National Institutes of Health.

### 4.4 Methodological Considerations for Future Research

To strengthen the quality of evidence in this field, future research would benefit from addressing specific challenges related to diagnostic heterogeneity and study design.

One significant hurdle identified is the variability in the definition of ‘end‐of‐life anxiety'. As noted during the review process, generic inclusion criteria may dilute observed treatment effects. We propose that future studies could be enhanced by adopting more precise diagnostic frameworks, such as those outlined in the **ICD‐11**. Specifically, differentiating between *Generalized Anxiety Disorder* and *Adjustment Disorders* or *Disorders specifically associated with stress* could help researchers recruit more homogeneous cohorts. Furthermore, establishing ‘Existential Distress' as a validated inclusion criterion may improve the targeting of interventions like psychedelic‐assisted therapy.

Additionally, the challenge of maintaining effective blinding in psychedelic research remains a concern, as suggested by the risk‐of‐bias assessment in the included studies. To mitigate the potential for expectancy bias, future trial designs should ideally incorporate ‘active placebos'—such as niacin or lower doses of the active compound, as utilized in some of the reviewed studies —to mimic physiological arousal without inducing the full psychedelic experience. Addressing these nosological and methodological factors may be crucial for facilitating high‐quality trials in this vulnerable population.

### 4.5 What This Study Adds

This systematic review provides an updated synthesis of current evidence on pharmacological interventions specifically targeting anxiety in end‐of‐life care, highlighting both traditional approaches and emerging treatments. Our findings suggest that benzodiazepines and opioids continue to represent important therapeutic options, although supported by limited empirical data, while psilocybin has emerged as a promising alternative, demonstrating rapid, sustained effects and a favorable safety profile. Nevertheless, significant methodological limitations and the restricted scope of available studies highlight considerable gaps in the evidence base, emphasizing the urgent need for further research in this area.

Future research should address critical unanswered questions, such as.

- How effective and safe are benzodiazepines and opioids specifically for anxiety, independent of their analgesic or sedative effects, in broader end‐of‐life populations beyond oncology?
- Could the promising effects observed with psilocybin in cancer patients generalize to individuals with other terminal illnesses, such as advanced cardiac or neurological conditions?
- What are the optimal dosing strategies, frequency of administration, and ideal timing for initiating psilocybin therapy to manage anxiety at the end of life?
- How do regulatory, cultural, and healthcare‐system barriers affect the feasibility and acceptability of implementing psilocybin‐based treatments in diverse global contexts?

Addressing these questions through robust future studies could significantly advance clinical practice and inform evidence‐based guidelines for managing anxiety in end‐of‐life care, enhancing both patient‐centered approaches and the quality of remaining life.

## 5 Conclusion

This systematic review suggests that distinct pharmacological strategies may be effective for different presentations of anxiety in end‐of‐life care: benzodiazepine‐opioid combinations appear beneficial for dyspnea‐related anxiety, while psilocybin shows promise for alleviating existential distress. However, these conclusions are drawn from a limited number of studies with varying methodologies. Consequently, these findings should be interpreted with caution. Future research is needed to validate these preliminary results, ideally prioritizing nosological precision aligned with ICD‐11 criteria and employing robust designs, such as active‐placebo controls, to minimize bias. Such advancements are essential to move toward evidence‐based guidelines for managing the complex psychological needs of terminally ill patients.

## Funding

No specific funding was received from public, commercial, or non‐profit organizations to support this research. The author B.F.C. acknowledges financial support through a medical residency in Psychiatry scholarship from the Ministry of Education of Brasil.

## Consent

Since this study is a systematic review of previously published literature and did not involve direct intervention or data collection from human participants by the authors, ethical approval and patient consent were not required.

## Conflicts of Interest

The authors declare no conflicts of interest.

## Supporting information


Supporting Information S1

## Data Availability

The data that support the findings of this study are available on request from the corresponding author. The data are not publicly available due to privacy or ethical restrictions.
