# Supplementary material for: Pharmacological Management of Anxiety in End‐of‐Life Care: A Systematic Review of Benzodiazepines, Opioids, and Psilocybin
Source: Hum Psychopharmacol. 2026 Jan 7;41(1):e70032. doi: 10.1002/hup.70032 (PMC12780303; doi:10.1002/hup.70032)
Supplement: Supplementary file 1 — Supporting Information S1 [file HUP-41-e70032-s001.docx]

**Appendix**

Specific approaches used for each search tool:

- PubMed:

(("End of life care" OR "terminal care" OR "hospice care" OR "terminally ill" OR "end-stage disease" OR "advanced disease") AND

("Anxiety" OR "anxiousness" OR "nervousness" OR "panic" OR "fear" OR "worry") AND

("pharmacological treatment" OR "drug therapy" OR "medication" OR "pharmacotherapy" OR "benzodiazepines" OR "antidepressants" OR "SSRIs" OR "SNRIs" OR "MAOIs" OR "tricyclic antidepressants" OR "antipsychotics" OR "anxiolytics" OR "sedatives" OR "tranquilizers") AND

("Placebo" OR "usual care" OR "standard care" OR "control group" OR "comparative") AND

("Efficacy" OR "effectiveness" OR "outcomes" OR "symptom relief" OR "symptom reduction" OR "quality of life" OR "QOL" OR "side effects" OR "adverse effects" OR "safety"))

- EMBASE:

('end of life care' OR 'terminal care' OR 'hospice care' OR 'terminally ill' OR 'end-stage disease' OR 'advanced disease') AND

('anxiety' OR 'anxiousness' OR 'nervousness' OR 'panic' OR 'fear' OR 'worry') AND

('pharmacological treatment' OR 'drug therapy' OR 'medication' OR 'pharmacotherapy' OR 'benzodiazepines' OR 'antidepressants' OR 'SSRIs' OR 'SNRIs' OR 'MAOIs' OR 'tricyclic antidepressants' OR 'antipsychotics' OR 'anxiolytics' OR 'sedatives' OR 'tranquilizers') AND

('placebo' OR 'usual care' OR 'standard care' OR 'control group' OR 'comparative') AND

('efficacy' OR 'effectiveness' OR 'outcomes' OR 'symptom relief' OR 'symptom reduction' OR 'quality of life' OR 'QOL' OR 'side effects' OR 'adverse effects' OR 'safety')

- Cochrane Library:

(("End of life care" OR "terminal care" OR "hospice care" OR "terminally ill" OR "end-stage disease" OR "advanced disease") AND

("Anxiety" OR "anxiousness" OR "nervousness" OR "panic" OR "fear" OR "worry") AND

("pharmacological treatment" OR "drug therapy" OR "medication" OR "pharmacotherapy" OR "benzodiazepines" OR "antidepressants" OR "SSRIs" OR "SNRIs" OR "MAOIs" OR "tricyclic antidepressants" OR "antipsychotics" OR "anxiolytics" OR "sedatives" OR "tranquilizers") AND

("Placebo" OR "usual care" OR "standard care" OR "control group" OR "comparative") AND

("Efficacy" OR "effectiveness" OR "outcomes" OR "symptom relief" OR "symptom reduction" OR "quality of life" OR "QOL" OR "side effects" OR "adverse effects" OR "safety")

- Clinicaltrials.gov:
  - Condition/disease: "Anxiety" OR "anxiousness" OR "nervousness" OR "panic" OR "fear" OR "worry"
  - Other terms: "End of life care" OR "terminal care" OR "hospice care" OR "terminally ill" OR "end-stage disease" OR "advanced disease"
  - Intervention/treatment: "pharmacological treatment" OR "drug therapy" OR "medication" OR "pharmacotherapy" OR "benzodiazepines" OR "antidepressants" OR "SSRIs" OR "SNRIs" OR "MAOIs" OR "tricyclic antidepressants" OR "antipsychotics" OR "anxiolytics" OR "sedatives" OR "tranquilizers"

**Table - Reports excluded with reasons**

| **Study** | **Reason for exclusion** | **Study** | **Reason for exclusion** |
| --- | --- | --- | --- |
| (Bruera et al., 1985) | Not anxiety study | (Kerr et al., 2012) | Not anxiety study |
| (Mazzocato et al., 1999) | Not anxiety study | (Daubert and Bolesta, 2014) | Study protocol only |
| (Fainsinger et al., 2000) | Not anxiety study | (Balduzzi et al., 2014) | Not anxiety study |
| (Chiu et al., 2001) | Not anxiety study | (Ng et al., 2014) | Not anxiety study |
| (Navigante et al., 2003) | Protocol of included study | (Mitchell et al., 2015) | Not anxiety study |
| (Muller-Busch et al., 2003) | Different design | (Mücke et al., 2015) | Review |
| (Kissane et al., 2004) | No intervention | (Song et al., 2016) | Not anxiety study |
| (Kohara et al., 2005) | Not anxiety study | (Simon et al., 2016) | Review |
| (Morita et al., 2005) | Not anxiety study | (Sullivan et al., 2017) | Not anxiety study |
| (Weschules et al., 2006) | Not anxiety study | (Van Esch et al., 2018) | Not anxiety study |
| (Laval and Paris, 2008) | Not anxiety study | (Currow et al., 2018) | Not anxiety study |
| (Rietjens et al., 2008) | Different design | (Higginson et al., 2020) | Not anxiety study |
| (Hardy, 2009) | Review | (Bajwah et al., 2020) | Review |
| (Mercadante et al., 2009) | Not anxiety study | (Alderman et al., 2020) | Not anxiety study |
| (Rosengarten et al., 2009) | Not anxiety study | (Strang et al., 2021) | Not anxiety study |
| (Porzio et al., 2010) | Not anxiety study | (Atkin et al., 2023) | Ongoing study |
| (Moraska et al., 2010) | Not anxiety study |  | |

Tab. 2 - Table with all the studies excluded after two researchers independently assessed for eligibility.

**References**

Alderman, B., Webber, K., Davies, A., 2020. An audit of end-of-life symptom control in patients with corona virus disease 2019 (COVID-19) dying in a hospital in the United Kingdom. Palliat Med 34, 1249–1255. https://doi.org/10.1177/0269216320947312

Atkin, N., Philip, J., Ftanou, M., Krishnasamy, M., Moncur, D., Wong, A., Grobler, A., Agar, M., Currow, D., Le, B., 2023. A Randomised, Double-blind, Placebo-controlled Feasibility Study of Oral Lorazepam for Symptoms of Anxiety in People with Advanced Life-limiting Illness. Palliative Med. 37, 102. https://doi.org/10.1177/02692163231172891

Bajwah, S., Oluyase, A.O., Yi, D., Gao, W., Evans, C.J., Grande, G., Todd, C., Costantini, M., Murtagh, F.E., Higginson, I.J., 2020. The effectiveness and cost-effectiveness of hospital-based specialist palliative care for adults with advanced illness and their caregivers. Cochrane Database of Systematic Reviews 2020. https://doi.org/10.1002/14651858.CD012780.pub2

Balduzzi, S., Mantarro, S., Guarneri, V., Tagliabue, L., Pistotti, V., Moja, L., D’Amico, R., 2014. Trastuzumab-containing regimens for metastatic breast cancer. Cochrane Database of Systematic Reviews 2021. https://doi.org/10.1002/14651858.CD006242.pub2

Bruera, E., Roca, E., Cedaro, L., Carraro, S., Chacon, R., 1985. Action of oral methylprednisolone in terminal cancer patients: a prospective randomized double-blind study. Cancer treatment reports 69, 751‐754.

Chiu, T.-Y., Hu, W.-Y., Lue, B.-H., Cheng, S.-Y., Chen, C.-Y., 2001. Sedation for Refractory Symptoms of Terminal Cancer Patients in Taiwan. Journal of Pain and Symptom Management 21, 467–472. https://doi.org/10.1016/S0885-3924(01)00286-X

Currow, D., Louw, S., Hill, J., Fazekas, B., Clark, K., Davidson, P., Mcdonald, C., Sajkov, D., Mccaffrey, N., Doogue, M., et al., 2018. Sertraline in symptomatic chronic breathlessness: a double blind, randomised trial. European respiratory journal 52. https://doi.org/10.1183/13993003.congress-2018.PA2047

Daubert, E., Bolesta, S., 2014. Effect of lorazepam versus morphine on quality of life in hospice patients with dyspnea and anxiety. Journal of the american pharmacists association 54, e193. https://doi.org/10.1331/JAPhA.2014.14511

Fainsinger, R.L., Waller, A., Bercovici, M., Bengtson, K., Landman, W., Hosking, M., Nunez-Olarte, J.M., deMoissac, D., 2000. A multicentre international study of sedation for uncontrolled symptoms in terminally ill patients. Palliat Med 14, 257–265. https://doi.org/10.1191/026921600666097479

Hardy, S.E., 2009. Methylphenidate for the treatment of depressive symptoms, including fatigue and apathy, in medically ill older adults and terminally ill adults. Am. J. Geriatr. Pharmacother. 7, 34–59. https://doi.org/10.1016/j.amjopharm.2009.02.006

Higginson, I.J., Wilcock, A., Johnson, M.J., Bajwah, S., Lovell, N., Yi, D., Hart, S.P., Crosby, V., Poad, H., Currow, D., Best, E., Brown, S., 2020. Randomised, double-blind, multicentre, mixed-methods, dose-escalation feasibility trial of mirtazapine for better treatment of severe breathlessness in advanced lung disease (BETTER-B feasibility). Thorax 75, 176–179. https://doi.org/10.1136/thoraxjnl-2019-213879

Kerr, C., Drake, J., Milch, R., Brazeau, D., Skretny, J., Brazeau, G., Donnelly, J., 2012. Effects of methylphenidate on fatigue and depression: a randomized, double-blind, placebo-controlled trial. Journal of pain and symptom management 43, 68‐77. https://doi.org/10.1016/j.jpainsymman.2011.03.026

Kissane, D.W., Grabsch, B., Love, A., Clarke, D.M., Blosch, S., Smith, G.C., 2004. Psychiatric disorder in women with early stage and advanced breast cancer: A comparative analysis. Aust. New Zealand J. Psychiatry 38, 320–326. https://doi.org/10.1111/j.1440-1614.2004.01358.x

Kohara, H., Ueoka, H., Takeyama, H., Murakami, T., Morita, T., 2005. Sedation for Terminally Ill Patients with Cancer with Uncontrollable Physical Distress. Journal of Palliative Medicine 8, 20–25. https://doi.org/10.1089/jpm.2005.8.20

Laval, G., Paris, A., 2008. Methylphenidate in palliative care in cancer patient: a double-blind randomised trial versus placebo. Bulletin du cancer 95, 241‐246. https://doi.org/10.1684/bdc.2008.0581

Mazzocato, C., Buclin, T., Rapin, C., 1999. The effects of morphine on dyspnea and ventilatory function in elderly patients with advanced cancer: a randomized double-blind controlled trial. Annals of oncology : official journal of the european society for medical oncology 10, 1511‐1514. https://doi.org/10.1023/a:1008337624200

Mercadante, S., Intravaia, G., Villari, P., Ferrera, P., David, F., Casuccio, A., 2009. Controlled Sedation for Refractory Symptoms in Dying Patients. Journal of Pain and Symptom Management 37, 771–779. https://doi.org/10.1016/j.jpainsymman.2008.04.020

Mitchell, G., Hardy, J., Nikles, C., Carmont, S., Senior, H., Schluter, P., Good, P., Currow, D., 2015. The Effect of Methylphenidate on Fatigue in Advanced Cancer: an Aggregated N-of-1 Trial. Journal of pain and symptom management 50, 289‐296. https://doi.org/10.1016/j.jpainsymman.2015.03.009

Moraska, A., Sood, A., Dakhil, S., Sloan, J., Barton, D., Atherton, P., Suh, J., Griffin, P., Johnson, D., Ali, A., et al., 2010. Phase III, randomized, double-blind, placebo-controlled study of long-acting methylphenidate for cancer-related fatigue: north Central Cancer Treatment Group NCCTG-N05C7 trial. Journal of clinical oncology 28, 3673‐3679. https://doi.org/10.1200/JCO.2010.28.1444

Morita, T., Chinone, Y., Ikenaga, M., Miyoshi, M., Nakaho, T., Nishitateno, K., Sakonji, M., Shima, Y., Suenaga, K., Takigawa, C., Kohara, H., Tani, K., Kawamura, Y., Matsubara, T., Watanabe, A., Yagi, Y., Sasaki, T., Higuchi, A., Kimura, H., Abo, H., Ozawa, T., Kizawa, Y., Uchitomi, Y., 2005. Efficacy and Safety of Palliative Sedation Therapy: A Multicenter, Prospective, Observational Study Conducted on Specialized Palliative Care Units in Japan. Journal of Pain and Symptom Management 30, 320–328. https://doi.org/10.1016/j.jpainsymman.2005.03.017

Mücke, M., Mochamat, M., Cuhls, H., Peuckmann-Post, V., Minton, O., Stone, P., Radbruch, L., 2015. Pharmacological treatments for fatigue associated with palliative care. Cochrane Database of Systematic Reviews 2020. https://doi.org/10.1002/14651858.CD006788.pub3

Muller-Busch, H.C., Andres, I., Jehser, T., 2003. Sedation in palliative care – a critical analysis of 7 years experience. BMC Palliat Care 2, 2. https://doi.org/10.1186/1472-684X-2-2

Navigante, A., Cerchietti, L., Cabalar, M., 2003. Morphine plus midazolam versus oxygen therapy on severe dyspnea management in the last week of life in hipoxemic advanced cancer patients. Medicina paliativa 10, 14‐19.

Ng, C.G., Boks, M.P.M., Roes, K.C.B., Zainal, N.Z., Sulaiman, A.H., Tan, S.B., De Wit, N.J., 2014. Rapid response to methylphenidate as an add-on therapy to mirtazapine in the treatment of major depressive disorder in terminally ill cancer patients: A four-week, randomized, double-blinded, placebo-controlled study. European Neuropsychopharmacology 24, 491–498. https://doi.org/10.1016/j.euroneuro.2014.01.016

Porzio, G., Aielli, F., Verna, L., Micolucci, G., Aloisi, P., Ficorella, C., 2010. Efficacy and safety of deep, continuous palliative sedation at home: a retrospective, single-institution study. Support Care Cancer 18, 77–81. https://doi.org/10.1007/s00520-009-0632-4

Rietjens, J.A.C., Van Zuylen, L., Van Veluw, H., Van Der Wijk, L., Van Der Heide, A., Van Der Rijt, C.C.D., 2008. Palliative Sedation in a Specialized Unit for Acute Palliative Care in a Cancer Hospital: Comparing Patients Dying With and Without Palliative Sedation. Journal of Pain and Symptom Management 36, 228–234. https://doi.org/10.1016/j.jpainsymman.2007.10.014

Rosengarten, O.S., Lamed, Y., Zisling, T., Feigin, A., Jacobs, J.M., n.d. Palliative Sedation at Home.

Simon, S.T., Higginson, I.J., Booth, S., Harding, R., Weingärtner, V., Bausewein, C., 2016. Benzodiazepines for the relief of breathlessness in advanced malignant and non-malignant diseases in adults. Cochrane Database of Systematic Reviews 2016. https://doi.org/10.1002/14651858.CD007354.pub3

Song, M., Ward, S., Lin, F., Hamilton, J., Hanson, L., Hladik, G., Fine, J., 2016. Racial differences in outcomes of an advance care planning intervention for dialysis patients and their surrogates. Journal of palliative medicine 19, 134‐142. https://doi.org/10.1089/jpm.2015.0232

Strang, P., Bergström, J., Lundström, S., 2021. Symptom Relief Is Possible in Elderly Dying COVID-19 Patients: A National Register Study. Journal of Palliative Medicine 24, 514–519. https://doi.org/10.1089/jpm.2020.0249

Sullivan, D., Mongoue-Tchokote, S., Mori, M., Goy, E., Ganzini, L., 2017. Randomized, double-blind, placebo-controlled study of methylphenidate for the treatment of depression in SSRI-treated cancer patients receiving palliative care. Psycho-oncology 26, 1763‐1769. https://doi.org/10.1002/pon.4220

Van Esch, H., Van Zuylen, L., Van Der Heide, A., Van Der Rijt, K., 2018. Scopolamine butyl given prophylactically for death rattle: A study protocol of a randomized double-blind placebo-controlled trial-the silence study, a first impression. Palliative Med. 32, 82–83. https://doi.org/10.1177/0269216318769196

Weschules, D.J., Maxwell, T., Reifsnyder, J., Knowlton, C.H., 2006. Are newer, more expensive pharmacotherapy options associated with superior symptom control compared to less costly agents used in a collaborative practice setting? Am J Hosp Palliat Care 23, 135–149. https://doi.org/10.1177/104990910602300211
